# Analysis of Mononuclear Phagocytes Disclosed the Establishment Processes of Two Macrophage Subsets in the Adult Murine Kidney

**DOI:** 10.3389/fimmu.2022.805420

**Published:** 2022-03-10

**Authors:** Qian Zhu, Jian He, Yangyang Cao, Xiaoli Liu, Wanyun Nie, Fei Han, Peng Shi, Xiao Z. Shen

**Affiliations:** ^1^ Department of Physiology and Department of Cardiology, The Second Affiliated Hospital, Zhejiang University School of Medicine, Hangzhou, China; ^2^ Department of Neurology, Zhejiang Hospital, Zhejiang University School of Medicine, Hangzhou, China; ^3^ Kidney Disease Center, The First Affiliated Hospital, Zhejiang University School of Medicine, Hangzhou, China; ^4^ Department of Cardiology, The Second Affiliated Hospital, and Institute of Translational Medicine, Zhejiang University School of Medicine, Hangzhou, China

**Keywords:** kidney, monocyte, dendritic cell, macrophage, CX3CR1

## Abstract

The interstitium of kidney involves a variety of components including resident immune cells, in particular mononuclear phagocytes. However, many proposed markers for distinguishing macrophages or dendritic cells are, in fact, shared by the majority of renal mononuclear phagocytes, which impedes the research of kidney diseases. Here, by employing a flow cytometry strategy and techniques of fate mapping, imaging and lineage depletion, we were able to demarcate renal monocytes, macrophages and dendritic cells and their subsets in mice. In particular, using this strategy, we found that even in steady state, the renal macrophage pool was continuously replenished by bone marrow-derived monocytes in a stepwise process, i.e., from infiltration of classical monocyte, to development of nonclassical monocyte and eventually to differentiation to macrophages. In mechanism, we demonstrated that the ligation of tissue-anchored CX3CL1 and monocytic CX3CR1 was required for promoting monocyte differentiation to macrophages in the kidney, but CX3CL1-CX3CR1 signaling was dispensable in monocyte infiltrating into the kidney. In addition to the bone marrow-derived macrophages, fate mapping in adult mice revealed another population of renal resident macrophages which were embryo-derived and self-maintaining. Thus, the dissecting strategies developed by us would assist in exploration of the biology of renal mononuclear phagocytes.

## Introduction

Entrenchment of resident macrophages in most tissues begins during embryogenesis from yolk-sac pro-macrophages and/or fetal liver-derived monocytes (1). An explicit example is microglia of the central nervous system. Microglia are exclusively derived from yolk sac and self-renewal can sustain their maintenance and function throughout lives, independent of the adult hematopoiesis (2). However, in some organs including gut, heart and lung, the resident macrophage pool is continuously replenished by bone marrow-derived monocytes in adults (1, 3–5), although the rate of renewal is widely different across organs. Deriving from the common monocyte progenitors (cMoP) in bone marrow (BM) (6), Ly6C^hi^ classical monocytes are the definitive precursors of many tissue mononuclear phagocytes (7). They routinely survey steady-state tissues (8) where they can further differentiate to monocyte-derived macrophages. Emerging evidence suggests that the embryo-derived self-maintaining macrophages (SM-MØ) coexist with their BM-derived counterparts in some tissues in adults (5, 9), but their proportions varies among organs. Thus, the major emerging challenges are to identify the distinctive subtypes of macrophages in their residing tissues and to understand the mechanisms underlying their establishment in specific tissues.

In many tissues, macrophages are phenotypically defined as F4/80^hi^, and conventional dendritic cells (cDCs) are CD11c^+^ MHC-II^hi^ in mice. However, it has been revealed that many of the proposed unique markers are, in fact, shared between macrophages, DCs and even monocytes. All cDCs are derived from the BM common DC progenitor (CDP), express the transcription factor ZBTB46, and arrive at the tissues *via* the blood stream (10). Further, they have a high turnover rate (hours to a few weeks), and thus are lack of embryonic input in adults (11). Seminal studies compared the transcriptomes of macrophages and DCs, shedding light on their signature pan-markers distinguishable from each other (12, 13). It has been proposed that in the occasion of inflammation, monocytes would locally differentiate into monocyte-derived DCs (moDCs) that express the surface markers CD11c, MHC-II, and Ly6C (14), but this viewpoint is recently challenged by a lineage-tracing study *in vivo* (15). Using a *Mafb*-driven Cre strain, Wu et al. demonstrated that Ly6C could not be used as a marker for either monocyte-derived macrophages or moDCs (15).

Adding to the complexity of myeloid cell discrimination, the majority of renal myeloid cells are F4/80^hi^CD11c^+^MHC-II^+^ (16). Some groups claimed that these cells were DCs (17), while others identified renal macrophages by a single marker F4/80 (18, 19). This inconsistent definition in the field has led to confusion and debate in the studies of renal macrophages and renal DCs (20, 21). To avoid inaccuracy, many researchers instead adopted the term “mononuclear phagocytes” to describe their research subjects in the kidney (22–25). Indeed, some tissue CD11c^+^MHC-II^+^ cells, e.g., small intestine CD103^–^CD11b^+^F4/80^+^CD11c^+^MHC-II^+^ cells and thioglycolate-elicited peritoneal CD11c^+^MHC-II^+^ cells, presented more macrophage signature than DC’s in cross-tissue transcriptome studies (12, 13); thus, the phenotypic definition of DCs, based on expression of MHC-II and CD11c, is not sufficient to identify tissue DCs. Thus, it is imperative to design a strategy to demarcate the renal mononuclear phagocytes and define their subsets.

## Materials and Methods

### Mice

*Zbtb46^gfp^ mice [027618], Cx3cr1* ^CreERT2^ mice [020940], *Rosa26-stop-TdTomato* mice [007914], *Cx3cr1* ^GFP/+^ mice [005582] and CD45.1^+^ C57BL/6 mice [002014] were from The Jackson Laboratory. All the mice are in C57BL/6 background. Normal C57BL/6 mice (CD45.2^+^) were purchased from Shanghai Research Center for Model Organisms. All mice used in this study without specific explanation were 8- to 12-week-old males. Mice were housed in a standard animal facility, with a 12-hr light/dark cycle, in specific-pathogen-free environment. All animal experiments were approved by the Institutional Animal Care and Use Committee at Zhejiang University (protocol ZJU20190135).

### Antibodies

The following fluorophore-conjugated antibodies were purchased from BioLegend or Thermo Fisher Scientific: anti-CCR2 (SA203G11, APC-0.5μg/10^6^ cells), anti-CD3 (145-2C11, FITC-0.25μg/10^6^ cells, APC-0.4μg/10^6^ cells), anti-CD11b (M1/70, FITC-0.1μg/10^6^ cells, PE-0.1μg/10^6^ cells, APC-0.25μg/10^6^ cells, Pacific Blue-0.1μg/10^6^ cells), anti-CD11c (N418, APC/Cyanine7-1μg/10^6^ cells, Pacific Blue-0.2μg/10^6^ cells), anti-CD19 (6D5, FITC-0.4μg/10^6^ cells, APC-0.2μg/10^6^ cells), anti-CD24 (M1-69, BV605-0.25μg/10^6^ cells), anti-CD45 (30-F11, FITC-0.1μg/10^6^ cells, APC-0.25μg/10^6^ cells, Pacific Blue-0.1μg/10^6^ cells, BV510-0.5μg/10^6^ cells), anti-CD45.1 (A20, APC-0.5μg/10^6^ cells), anti-CD45.2 (104, PE-0.5μg/10^6^ cells), anti-CD49b (DX5, FITC-1μg/10^6^ cells), anti-CD62L (MEL-14, PE-0.2μg/10^6^ cells), anti-CD64 (X54-5/7, FITC-0.2μg/10^6^ cells, PE-1μg/10^6^ cells), anti-CD103 (2E7, PE-0.25μg/10^6^ cells), anti-VCAM-1 (429MVCAM.A, Biotin-0.0625μg/10^6^ cells), anti-CD115 (AFS98, PE-0.25μg/10^6^ cells, PE/Cyanine7-0.0625μg/10^6^ cells), anti-F4/80 (BM8, APC-0.2μg/10^6^ cells, PE/Cyanine7-0.25μg/10^6^ cells, APC/Cyanine7-0.5μg/10^6^ cells), anti–I-A^b^ (AF6-120.1, PE-0.25μg/10^6^ cells, Alexa 488-0.1μg/10^6^ cells, APC/Cyanine7-0.25μg/10^6^ cells), anti-CX3CR1 (SA011F11, BV510-0.02μg/10^6^ cells), anti-CD88(20/70, APC-1μg/10^6^ cells), anti-XCR1(ZET, PE-0.25μg/10^6^ cells), anti-Ly6C (HK1.4, Pacific Blue-0.1 μg/10^6^ cells), anti-Ly6G (1A8, APC-0.25μg/10^6^ cells, FITC-0.2μg/10^6^ cells) and anti-CD26(H194-112, APC-1μg/10^6^ cells). For immunohistostaining, an anti-CD31 antibody (clone: MEC13.3, BioLegend) was used. For labeling intravascular leukocyte, each mouse was injected *i.v.* with 2.5 µg APC-conjugated anti-CD45 antibody (Clone 30-F11, BioLegend), and was harvested 2 min later.

### Reagents

FLT3 inhibitor AC220 (Adooq-bioscience) was administered to mice intragastrically in a dose of 10 mg/kg twice a day for two weeks. For depleting monocytes/macrophages, mice were injected i.v. with 10 µl/g liposome clodronate (Liposoma). For *in vivo* labeling monocytes, the stock 2.7% 0.5 μm fluorescent microspheres (Polysciences) was diluted 25 times by PBS, and 250 µl were injected i.v. to each mouse. For blocking CD11a, an anti-CD11a antibody (Clone: M17/4) (4mg/Kg) or an equivalent dose of isotype antibody was i.v. infused to mice one hour prior to perfusion and sacrifice. To estimate the proliferating rate, mice were injected *i.v.* with 10 mg/kg EdU (RiboBio) and were harvested 2 hr later.

### Renal Immune Cell Extraction and Flow Cytometry

Kidneys were isolated from mice after perfusion with cold PBS plus EDTA and were cut into small pieces. Each kidney was digested in 6 ml RPMI1640 (GIBICO) with 1.5 mg/ml collagenase IV (Worthington) and 25 u/ml DNase I (Sigma) for 30 min at 37°C with gentle shaking. After digestion, cells were passed through a 70-μm strainer (BD) and were then subject to gradient centrifugation by using 72% and 36% Percoll (GE Healthcare). Immune cells were enriched at the 72%/36% interface and were collected for flow cytometry analyses (dead cells were at the bottom so they were not included). Samples were analyzed with a 3-laser flow cytometer (Agilent Novocyte) and data were processed with FlowJo (v10.1). Renal macrophages were purified by cell sorting with a BD SORP ARIA II.

### Histology

Kidneys were fixed overnight at 4°C in paraformaldehyde, dehydrated in phosphate buffer containing 30% sucrose overnight at 4°C and then frozen on dry ice. Frozen sections (30 µm thick) were cut on a cryostat at -20°C (CM3050S; Leica), rehydrated in PBS, and permeabilized with PBS supplemented with 1% Triton X-100 (Sigma-Aldrich). Afterwards, the sections were blocked for 2 hr at room temperature with blocking buffer which contained 5% donkey serum in PBS. The sections were then incubated with primary antibodies in antibody dilution buffer (0.5% Triton X-100 and 1% BSA in PBS) overnight at 4°C, followed by incubation with secondary donkey antibodies in antibody dilution buffer for 2 hr at room temperature in the dark. After mounting on glass slides, stained sections were viewed under confocal microscope equipped with 10x/0.45, 20x/0.8, 40x/0.95 NA Plan-Apochromat objective (Carl Zeiss LSM 900). Images were displayed as maximum-intensity projections of 21 µm thick Z stacks recorded in 15 sections, and analyzed with the ZEN system software.

### *In Vitro* Phagocytosis Assay

The ways of kidney digestion and single cell preparation were mentioned above. Renal single cells were co-incubated with 0.03% 0.5 μm fluorescent latex beads (Polysciences) in DMEM supplemented with 1% FBS at 37° for 6 hr. After that, cells were washed and subject to Percoll enrichment for immune cells and flow cytometry analyses.

### Bone Marrow Transplantation

For competitive BM transplantation experiment, BM was obtained from 8-week-old *Cx3cr1* ^+/+^ and *Cx3cr1* ^GFP/GFP^ mice which were both in C57BL/6 CD45.2^+^ background. Nucleated cells were counted, and the BM was resuspended at a concentration of 2×10^7^/ml. Recipient mice were 8-week-old CD45.1^+^ C57BL/6 mice which were irradiated with 950 rads and then were immediately *i.v.* reconstituted with 2×10^6^ mixture of BM cells in a ratio of 3:1 between *Cx3cr1* ^GFP/GFP^ and *Cx3cr1* ^+/+^ background. To make torso-protected BM chimeric mice, CD45.2^+^ C57BL/6 recipients were anesthetized and positioned beneath a 5-mm-thick lead shield that only covered the torso, resulting in recipients receiving irradiation on exposed head and all four limbs. After irradiation, mice were reconstituted with 2×10^6^ CD45.1^+^ C57BL/6 BM cells.

### Monocyte Purification, Labeling and Adoptive Transfer

Classical monocytes were purified from bone marrow of C57BL/6 mice by a monocyte isolation kit (Stemcell Technologies, #19861)which adopts a negative selection strategy. 1.5x10^6^ CFSE (BioLegend)-labeled monocytes were adoptively transferred *i.v.* to naive 8-week-old C57BL/6 mice. In the indicated days after transfer, the kidneys of the recipients were collected and analyzed.

### Monocyte *In Vitro* Differentiation

For co-culture of monocytes and renal epithelial cell, freshly purified classical monocytes were seeded into a 96-well flat-bottom cell culture plate (Cat#3599, Corning) pre-cultured with a monolayer of HK2 cells (ATCC) or primary murine renal epithelial cells which was in 100% confluence. Cells were co-cultured in DMEM supplemented with 1% FBS in the presence of or without 7.5 μM CX3CR1 antagonist AZD8797 (MCE). After 20 hr, monocytes were harvested for flow cytometry analysis and they were distinguished from HK-2 cells by CD11b staining. To examine the role of anchored CX3CL1, high-adsorption 96-well plates (Cat#9018, Corning) were pre-coated with 20 μg/ml either recombinant mouse CX3CL1 (BioLegend) or BSA (Sigma) at 4°C overnight. After removing the coating solution, freshly purified monocytes were seeded into the plate in a density of 1.5×10^5^ per well in DMEM supplemented with 1% FBS. After a 10-hr co-incubation, cells were subject to flow cytometry analysis. For trans-well culture, epithelial cells were cultured to 70% confluence in the lower layer of a 24-well plate (Labselect) when 1×10^5^ monocytes were seeded onto the upper layer separated by a 0.4 μm insert. The monocytes were analyzed 10 hr later.

### Primary Culture of Murine Tubular Epithelial Cells

Kidneys were collected from C57BL/6 mice (3- to 5-week-old males). Cells were isolated from mice after perfusion with cold PBS plus EDTA and were cut into small pieces. Each kidney was digested in 6 ml RPMI1640 (GIBICO) with 1.5 mg/ml collagenase IV (Worthington) and 25 μl/ml DNase I (Sigma) for 30 min at 37°C with gentle shaking. After digestion, cells were filtered through a 100-µm strainer (BD). Cell suspensions were cultured in RPMI 1640 supplemented with 10% fetal bovine serum, 20 ng/ml Epidermal Growth Factor (Peprotech), 1× ITS (Insulin, Transferrin and Selenium, Gibco), and 1% penicillin-streptomycin (Corning) at 5% CO2 and 37 °C. When cell confluence reached 70%, the flowing cells were washed out and media were changed for the attached epithelial cells for another 48 hr.

### Tamoxifen Treatment

Tamoxifen (MCE) was dissolved in corn oil at a concentration of 15 mg/ml. To induce Cre expression in adult mice, mice were injected *i.p.* with 75 μg/g tamoxifen for 5 consecutive days. For labeling at P0, each newborn pup was given *i.p.* with 0.75 mg tamoxifen.

### Statistics

Statistical analysis was performed with Prism 6.0 (GraphPad). Data are presented as mean ± SEM. Paired and unpaired Student’s t tests and One-way ANOVA with *post hoc* tests were used. All statistical tests were two tailed, and P values of <0.05 were considered significant.

## Results

### Discriminate Renal Mononuclear Phagocytes

**Figure 1:**
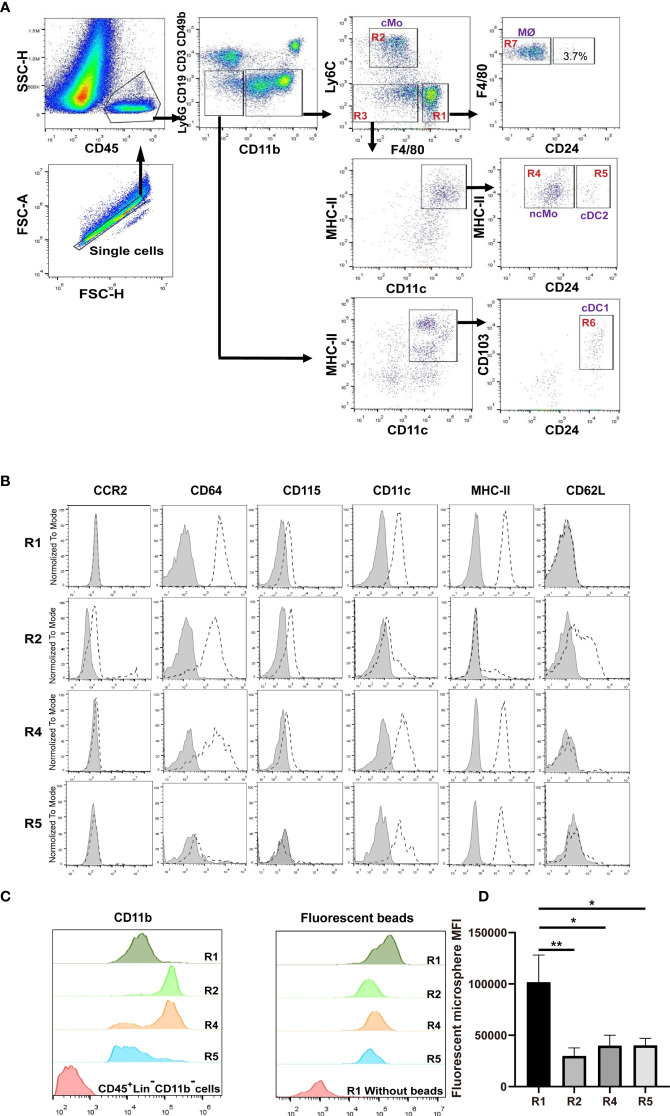
Distinguish subsets of mononuclear phagocytes in adult murine kidneys. **(A)** Immune cells were first enriched with Percoll gradient centrifugation which also helped separation from dead cells. The gating strategy for discriminating renal mononuclear phagocyte subsets is shown. cMo, classical monocytes; ncMo, nonclassical monocytes; MØ, macrophages; cDC1, classical dendritic cells 1; cDC2, classical dendritic cells 2. **(B)** Renal samples were stained with antibodies against the indicated markers (dot line) or related isotype control antibodies (shaded). The expression levels of the indicated markers on R1, R2, R4 and R5 subsets are shown (representative of n=5 per group). **(C)** The relative expression levels of CD11b were compared between R1, R2, R4 and R5 subsets (representative of n=5 per group). **(D)** Kidneys were digested and single cell suspension was prepared. Cells were then co-incubated with 0.5 μm fluorescent latex beads at 37° for 6 hours. The abilities in intake of microspheres by R1, R2, R4 and R5 cells were compared (representative of 3 independent experiments). All error bars indicate mean ± SEM. *P* values were calculated using One-way ANOVA test. *P < 0.05, **P < 0.01.

We devised a gating strategy in order to discriminate renal mononuclear phagocytes from kidneys of 8-12 weeks old mice. We excluded cells from T cell (CD3e), B cell (CD19), NK cell (CD49b) and neutrophil (Ly6G) lineages (**Figure 1A**). Analysis of CD45^+^CD11b^+^ cells using antibodies against F4/80 and Ly6C gave rise to three major populations, namely R1 (F4/80^hi^Ly6C^lo^), R2 (F4/80^lo^Ly6C^hi^ and R3 ((F4/80^neg-lo^Ly6C^lo^) (**Figure 1A**), among which R1 constituted the majority of the CD11b^+^ cells. The R1 cells were CD11b^lo^CD115^+^CCR2^—^ (**Figures 1B, C**), a phenotype resembling resident macrophages of other organs/tissues (26, 27). They were also highest in expressing the pan-macrophage marker CD64 (**Figure 1B**) (12). Besides, the R1 cells were most efficient in phagocytosis of latex beads *ex* vivo (**Figure 1D**).

**Figure 2:**
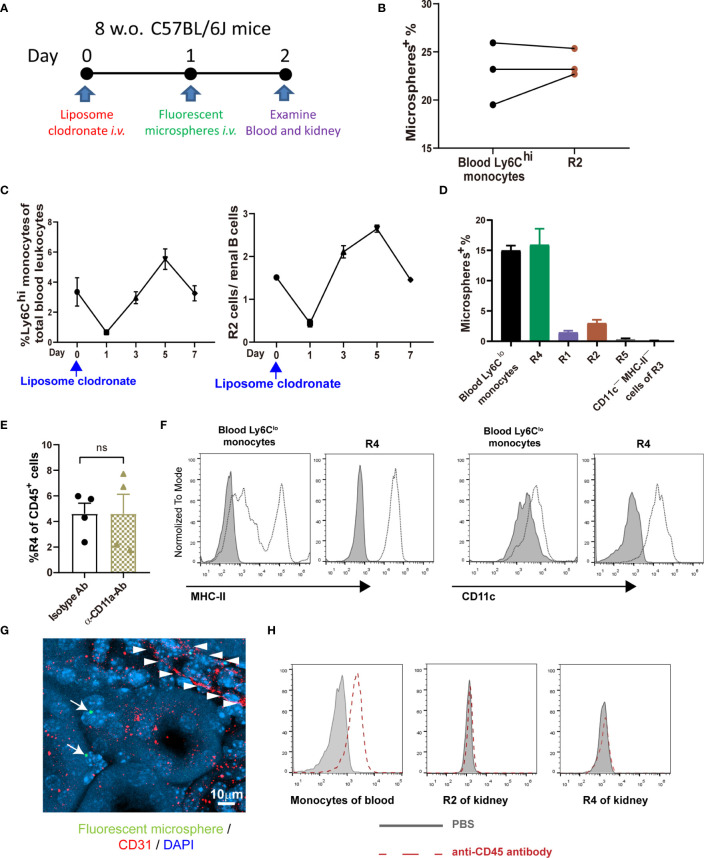
Defining the intrarenal monocyte subsets. **(A)** Working diagram of labeling and tracing classical monocytes. **(B)** The labeling rates of fluorescent microspheres in blood classical monocytes and renal R2 cells. Each dot represents the data derived from one mouse. **(C)** The depletion and recovery patterns of blood classical monocytes and renal R2 cells after liposome clodronate treatment (n=3-6 per group). **(D)** The labeling rates of fluorescent microspheres in blood nonclassical monocytes and the indicated renal mononuclear phagocyte subsets (n=3 per group). **(E)** One hour prior to perfusion, mice were *i.v.* treated with either an anti-CD11a antibody or an isotype control antibody. The yields of R4 cells were compared after extracting the kidneys. ns, not significant. **(F)** Expression of MHC-II and CD11c on blood Ly6C^lo^ nonclassical monocytes and on renal R4 cells were compared. The shaded is isotype antibody staining (representative of n=4 per group). Data were derived from 2 or more independent experiments. **(G)** A confocal image of microsphere-labeled nonclassical monocytes (arrow) in the interstitium of kidney. Arrowheads indicate the contour of a blood vessel. **(H)** The CD45 labeling efficiency of total blood monocytes and renal R2 and R4 monocytic cells after a fluorescently labeled anti-CD45 antibody was given *i.v.* 2 min before euthanasia (representative of n=3 per group). Data are representative of at least two independent experiments. All error bars indicate mean ± SEM.

Classical monocytes routinely survey steady-state tissues (8). Characterizing the cells in R2 showed that besides highly expressing Ly6C, they were CD115^+^CD11b^hi^CCR2^+^CD64^int^ CD62L^+^ (**Figures 1B, C**), consistent with the phenotype of blood classical monocytes. To verify that R2 were the real classical monocytes infiltrating into renal parenchyma, we adopted a strategy by sequential treatment of liposome clodronate and fluorescent microspheres, which could label blood classical monocytes (**Figure 2A**) (28). Without liposome clodronate pre-treatment, intravenous injection of inert fluorescent microspheres would preferentially label Ly6C^lo^ nonclassical monocytes over Ly6C^hi^ classical monocytes (28). Administration of liposome clodronate depleted both classical and nonclassical monocytes (28). However, since classical monocytes are continuously generated from the BM and they are the progenitors of nonclassical monocytes (29), they will reappear in the blood one day after liposome clodronate treatment when nonclassical monocytes are still absent. During this window, classical monocytes could be labeled with the fluorescent microspheres. Thus, One day after liposome clodronate treatment, we *i.v*. injected the mice with microsphere; after another 24 hours, we examined the blood and kidneys of the recipients (**Figure 2A**). It showed that the labeling rate of R2 was close to that of blood classical monocytes (**Figure 2B**). In addition, only the pattern of depletion and recovery of renal R2 cells after liposome clodronate treatment was in accordance with that of blood classical monocytes; in contrast, R1 had a much delayed recovery pattern after depletion (**Figure 2C** and **Figure S1**). We used renal B cells as an internal control here since they are unaffected by liposome clodronate. These data demonstrate that the R2 represent the classical monocytes in the kidney. Most of the R3 cells expressed both CD11c and MHC-II, resembling DCs, but the CD11c^+^MHC-II^+^ cells were divergent in CD24 expression (**Figure 1A**). We named the CD24^—^ cells R4 and CD24^+^ cells R5. We noticed that only the R4 cells, but not the R1, R2, R5 or the CD11c^—^MHC-II^—^ cells of R3, were labeled as efficiently as blood nonclassical monocytes when examined 24 hr post microsphere labeling (without liposome clodronate pre-treatment) (**Figure 2D**), suggesting that the R4 may represent nonclassical monocytes in the kidneys. Moreover, these cells were Ly6C^lo^CD115^+^ CD11b^hi^CCR2^—^CD64^int^CD62L^—^ in phenotype (**Figure 1B**), in line with nonclassical monocytes. In resting state, nonclassical monocytes were known to continuously crawl on the lumen side of endothelial cells of blood vessels (30). We thus wondered whether the observed R4 cells actually were attaching inside the vasculature but were not residing in the renal interstitium. LFA1(CD11a)-ICAM ligation is required for the crawling of Ly6C^lo^ monocytes on the vessel wall (31). However, blocking CD11a prior to perfusion did not reduce the frequency of R4 (**Figure 2E**). Also, when comparing to blood Ly6C^lo^ monocytes (**Figure S2**), R4 cells expressed higher levels of CD11c and MHC-II (**Figure 2F**), implying they resided in microenvironment distinctive from blood. To further distinguish the anatomical position of R4 cells in the kidney, we performed immunohistostaining after *i.v*. fluorescent microsphere labeling. This showed that almost all microsphere-labeled cells (microsphere in proximity to DAPI^+^ nucleus), were indeed in the renal interstitium but not in the intraluminal side of CD31^+^ blood vessels or renal tubules which were readily identifiable by their tubular structure (**Figure 2G**). To be sure that the renal tissue monocytes evaluated were not mixed with blood monocytic counterparts that remained within the vasculature of kidneys as they were processed, we *i.v.* injected a labeled anti-CD45 antibody 2 min before euthanasia. In contrast to blood monocytes which were almost completely stained by this *i.v*. infused anti-CD45 antibody, the R2 (classical monocytes) and R4 cells of the kidneys were unstained (**Figure 2H**). Cumulatively, R4 appears a group of nonclassical monocytes in renal parenchyma.

**Figure 3:**
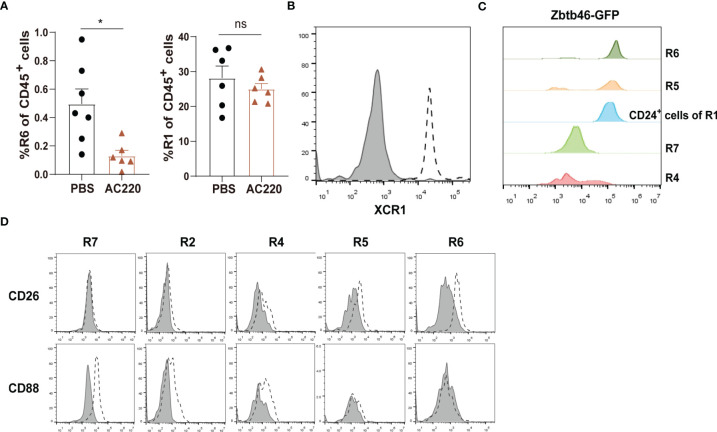
Distinguishing macrophages from classical DCs in the kidney **(A)** Treating C57BL/6 mice with Flt3 inhibitor AC220 for 2 weeks significantly reduced the cell number of R6 cDC1s but had little impact on R1 cells. *P < 0.05; ns: not significant. *P* values were calculated using a two-tailed unpaired t-test. **(B)** Renal samples were stained with an anti-XCR1 antibody (dot line) or an isotype antibody (shaded). The R6 cells were examined (representative of *n=*3 per group). **(C)** Zbtb46 expression was evaluated for renal mononuclear phagocyte subsets by GFP fluorescent intensity derived from *Zbtb46* ^GFP^ *^/+^* mice (representative of *n=*3 per group). **(D)** Renal samples were stained with antibodies against the indicated markers (dot line) or related isotype control antibodies (shaded). The expression levels of the indicated markers on R7, R2, R4, R5 and R6 subsets are shown (representative of n=3 per group). Data are representative of at least two independent experiments. All error bars indicate mean ± SEM.

R1 cells highly express both CD11c and MHC-II, two markers widely used for indicating DCs (**Figure 1B**). To distinguish DCs in the kidney, we first relied on other markers which could more faithfully delineate potential DCs. The classical DCs (cDCs) can be divided into two subsets, cDC1 and cDC2. In nonlymphoid tissue, cDC1s are CD103^+^CD11b^—^ and cDC2s are CD103^—^CD11b^+^, but both of them express CD24; in addition, XCR1 is exclusively expressed by cDC1 (32). We noticed that in the CD45^+^Lin^—^CD11b^—^ population, there was a subgroup of CD11c^+^MHC-II^+^ cells, denoted as R6, expressing both CD103 and CD24 (**Figure 1A**). Treatment with Flt3 blocker for 2 weeks significantly reduced the cell number of R6 but not those of R1, indicating that the R6 were cDC1s (**Figure 3A**) (33, 34). Their cDC1 identity was further confirmed by their XCR1 expression (**Figure 3B**). The R5 cells mentioned above were CD11b^+^CD11c^+^MHC-II^+^CD24^+^, resembling cDC2s (**Figure 1A**). Intriguingly, a minority (about 4%) of R1 cells also expressed CD24 (**Figure 1A**), implying that they were possibly admixed with DCs. To distinguish cDCs from other myeloid cells, we employed *Zbtb46* ^GFP/+^ mice which faithfully label cDC lineage cells with GFP (35). R6 cells uniformly highly expressed Zbtb46, confirming their cDC1 identity (**Figure 3C**). Most of R5 cells were GFP+, representing the genuine cDC2s. In terms of R1, the CD24^—^ cells were uniformly negative for GFP expression, whilst the CD24^+^ subpopulation was positive for GFP expression. In conclusion, the Lin^—^CD11b^lo^F4/80^hi^CD24^—^ cells (denoted as R7) were not contaminated with DCs. Recent reports have indicated that CD88 and CD26 could be used for discrimination of monocytes/macrophages from cDCs, as the monocytes/macrophages were CD88^+^ (36) while the cDCs were CD26^+^ (37). Consistent with these patterns, the renal R7 cells were CD88^+^CD26^—^, while both R5 and R6 cells were CD88^—^CD26^+^ (**Figure 3D**). We thereafter focused on R7 for studies on renal macrophages. The patterns of discrete markers for distinguishing renal mononuclear phagocytes were summarized in **Table 1**.

**Table 1:** Surface marker expression on the subsets of renal mononuclear phagocytes.

	CD11b	CCR2	CD64	CD115	CD11c	MHC-II	CD62L	CD88	CD26
**R2**	++	+	+	+	–	–	+	+	–
**R4**	++	–	+	+	++	+	–	+	–
**R5**	+	–	–	–	++	+	–	–	+
**R6**	–	–	–	–	++	+	+/–	–	+
**R7**	+	–	++	+	+	+	–	+	–

### Assessment of the Subsets of Renal Resident Macrophages

**Figure 4:**
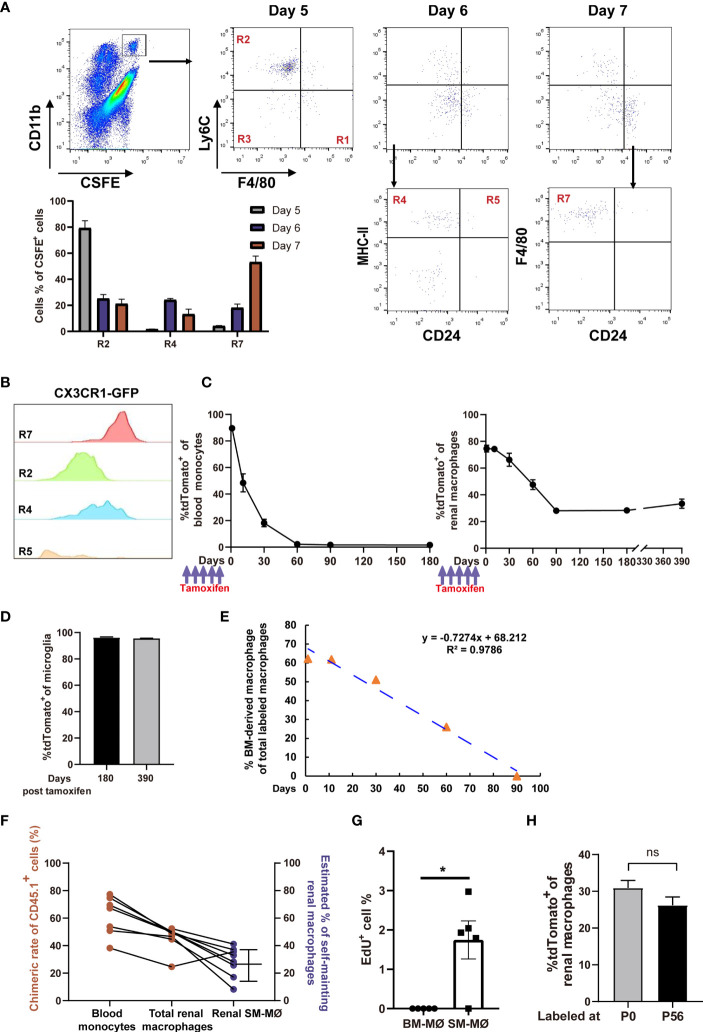
Fate-mapping reveals that mouse kidneys consist of embryo-derived self-maintaining macrophages and BM-derived macrophages. **(A)** CFSE-labeled classical monocytes were adoptively transferred *i.v.* into naive mice, and their fates were traced in the kidneys (representative of n=5 per group). The quantification of CFSE^+^ cells in the R2, R4 and R7 gates on Days 5, 6 and 7 post transfer was shown. **(B)** CX3CR1 expression, as indicated by GFP, was evaluated in the renal mononuclear phagocyte subsets of *Cx3cr1* ^GFP^ *^/+^* mice (representative of 3 independent experiments). **(C, D)** 8-weeks old *Cx3cr1^CreER/+^:R26* ^Td^ mice were *i.p.* treated with tamoxifen. The percentages of tdTomato^+^ cells among **(C)** blood monocytes, renal macrophages and **(D)** brain microglia(CD45^+^CD11b^lo^) were traced on the indicated time points. (For Days 0-180, n=4 per group; For Day 390, *n*=2 per group). **(E)** First-order elimination kinetics of the BM-derived renal macrophages. **(F)** Five months after torso-shielded BM transplantation, the chimeric rates of donor bone marrow-derived cells (CD45.1^+^) in blood monocytes and total renal macrophages are shown (ruby dots). The estimated frequencies of SM-MØ were shown in blue dots. **(G)** The percentages of EdU^+^ cells in the tdTomato^—^ BM-derived macrophages and tdTomato^+^ SM-MØ 2 hr after EdU infusion into the *Cx3cr1^CreER/+^:R26* ^Td^ mice pre-treated with tamoxifen 3 months ago. *P* values were calculated using a two-tailed paired t-test. *P < 0.05. **(H)** *Cx3cr1^CreER/+^:R26* ^Td^ mice were injected with tamoxifen at P0 or P56. The percentages of tdTomato^+^ cells among renal macrophages were examined 3 months later. (*n*=4 for P0 and *n*=6 for P56). ns: not significant. Data were derived from 2 or more independent experiments.

Previous studies showed that under stresses induced by kidney injury (38) or macrophage depletion (19), monocytes were recruited and reconstructed the renal macrophage pool. Identification of renal distribution of monocyte subsets in steady state made us to interrogate whether monocytes continuously differentiate into renal macrophages in a resting condition. To this end, we intravenously transferred naïve mice with CFSE-labeled BM-selected monocytes, over 95% of which were Ly6C^hi^ classical monocytes (**Figure S3**). Lineage tracing showed that after transfer, the labeled monocytes first appeared in the R2 of kidney, further verifying that R2 were directly derived from blood classical monocytes (**Figure 4A**). Afterwards, most of the transferred cells took stepwise differentiation from classical monocytes to renal macrophages, as they sequentially accumulated in the R4 and then turned into R7 (**Figure 4A**). In addition, a few of the transferred cells appeared in R5 and the unidentified CD11c^—^MHC-II^—^ subset of R3, suggesting multi-direction differentiation potential of classical monocytes in the kidney. Thus, it appeared that blood monocytes could routinely infiltrate and contribute to the renal macrophage pool.

A recent study suggests that renal resident macrophages encompass both BM-derived macrophages and embryo-derived macrophages which are self-maintaining (19). CX3CR1 is expressed diversely in monocytes and tissue macrophages (30). In the kidney, macrophages (R7) were homogenously expressing CX3CR1 and they are the highest in CX3CR1 expression among mononuclear phagocyte subsets (**Figure 4B**). To discriminate SM-MØ from BM-derived macrophages in the kidney and to estimate their frequencies by the flow cytometry scheme developed by us, we crossed a mouse line of tamoxifen-inducible *Cx3cr1* ^CreERT2^ controlled by the endogenous *Cx3cr1* promoter with *Rosa26-stop-tdTomato* r reporter mice (termed *Cx3cr1* ^CreER/+^ *:R26* ^Td^). Tamoxifen was first injected into 8-week old mice, and 1 day post tamoxifen treatment, the majority of CD115^+^CX3CR1^+^CD11b^+^ blood monocytes (89.6 ± 1.5%, mean ± SEM) and renal R7 macrophages (74.57 ± 4.45%) turned tdTomato^+^ (**Figure 4C**). Time-course analysis revealed that by 2 months, blood monocytes completely lost tdTomato expression, reflecting their continuous replacement by BM progenitors. Renal macrophages (R7) lost tdTomato^+^ cells in a slower pattern, and by 3 months, about 28.1 ± 5.27% cells retained tdTomato expression (**Figure 4C** and **Figure S4**). However, from then on, the mice did not lose tdTomato^+^ macrophages any more since there were still 28.35 ± 1.89% and 33.35 ± 4.88% macrophages being tdTomato^+^ when examined 180 days and even 390 days after labeling, respectively (**Figure 4C**). In contrast, on Day 180 and Day 390 after labeling, tdTomato^+^ cells respectively accounted for 96.28 ± 0.41% and 95.51 ± 0.21% of microglia, the turnover of which depends on self-renewal (**Figure 4D**) (39). Of note, 3 months after tamoxifen treatment, no other population except R7 contained tdTomato^+^ cells, implying that only a subset of macrophages but no other renal mononuclear phagocytes were self-maintaining. Estimated based on the First-order elimination kinetics, the residential half-life of BM-derived macrophages in the kidney was approximately 46.9 days (**Figure 4E**).

The frequency of renal SM-MØ estimated by our gating and fate mapping strategies was about 37% after considering the initial labeling rate (**Figure 4C**), which was much lower than ~60% claimed by a recent study using *ihCD59* mice (19). To estimate the frequency of renal SM-MØ by another independent means, we made chimeras by transplantation of CD45.1^+^ donor BM to CD45.2^+^ mice. To avoid disrupting kidney homoeostasis, we performed torso-shielded irradiation to protect the kidneys. Based on the facts that a 2-month period is required for a full establishment of transferred BM and a 3-month period is required for a complete replacement of BM-derived renal macrophages, we examined the blood monocytes and renal macrophages 5 months after BM transplantation. We could deduce the ratio of SM-MØ in the renal macrophage pool of each transplanted recipient based on its chimeric rates of blood monocytes and total renal macrophages (**Figure S5**). This led to an estimate of 28.2 ± 4.9% (**Figure 4F**), lower but close to the estimate derived from *Cx3cr1* ^CreER/+^ *:R26* ^Td^ mice. An EdU incorporation assay revealed that in steady state, the SM-MØ had about 1.8% cells in proliferation while there was barely any proliferating BM-derived macrophages in the kidney (**Figure 4G**).

To investigate whether the SM-MØ observed in the adult kidney had embryo origin, we injected the newborn *Cx3cr1* ^CreER/+^ *:R26* ^Td^ mice with tamoxifen at P0. When they were examined 90 days later, tdTomato^+^ exhibited about 30.93 ± 2.02% of total macrophages, close to the SM-MØ frequency estimated by labeling in adulthood (P56) (**Figure 4H**). Thus, adult murine kidneys contain two macrophage subsets, *i.e.*, embryo-derived SM-MØ and BM-derived macrophages, with the latter constituting the majority of resident macrophages.

### Differentiation of Classical Monocytes to Renal Macrophages Requires CX3CL1-CX3CR1 Signaling

**Figure 5:**
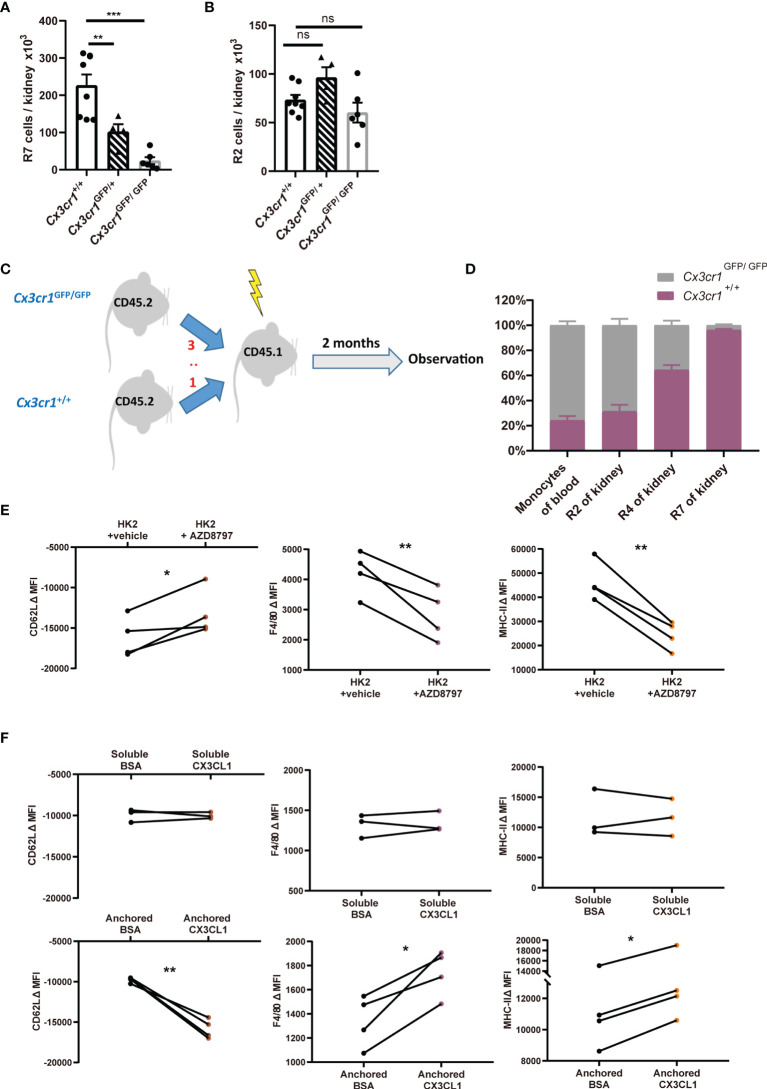
CX3CR1-dependent stepwise differentiation from classical monocytes to renal macrophages. **(A, B)** Quantification of renal macrophages (R7) **(A)** and classical monocytes (R2) **(B)** in *Cx3cr1* ^+/+^, *Cx3cr1* ^GFP/+^ and *Cx3cr1* ^GFP/GFP^ mice in steady state. *P* values were calculated using one-way ANOVA with Tukey’s multiple-comparisons testing. **(C)** Lethally irradiated CD45.1 mice were transplanted with a chimeric 3:1 BM mix from CD45.2 *Cx3cr1* ^GFP/GFP^ and *Cx3cr1* ^+/+^ donors. **(D)** Eight weeks after transplantation, the ratios of *Cx3cr1* ^+/+^ *vs. Cx3cr1* ^GFP/GFP^ among CD45.2^+^ blood monocytes, renal classical monocytes (R2), renal nonclassical monocytes (R4) and renal macrophages (R7) are shown. (*n*=4 per group from 2 independent experiments). **(E, F)** The changes of mean fluorescent intensities (ΔMFI) of surface CD62L, F4/80 and MHC-II on classical monocytes after *ex vivo* culture. **(E)** Monocytes were co-incubated with renal epithelial HK-2 cells in the presence of vehicle or AZD8797, a CX3CR1 blocker. **(F)** monocytes were cultured in the presence of soluble (Upper panels) or anchored bovine serum albumin (BSA) or recombinant CX3CL1 (Lower panels). *P* values were calculated using a two-tailed paired t-test. *P < 0.05, **P < 0.01, ***P < 0.005. ns: not significant. All error bars indicate mean ± SEM.

CX3CL1, the sole ligand of CX3CR1, is expressed in kidney glomerular and tubular epithelial cells (40). Many studies suggested that CX3CL1 functions as a chemokine for recruiting monocytes into the kidney under stress (19, 41). The observation that (I) there exist interstitial monocytes in the kidney (**Figures 2E–H**) and (II) macrophages are the highest in CX3CR1expression among renal mononuclear phagocyte subsets (**Figure 4B**) prompted us to investigate the role of CX3CR1 in the establishment of renal monocytes and macrophages. We first studied the CX3CR1-deficient *Cx3cr1* ^GFP/GFP^ mice which have a GFP sequence replacing the coding exon of the *Cx3cr1* gene. Compared to *Cx3cr1^+/+^* littermates, *Cx3cr1* ^GFP/GFP^ mice had markedly fewer R7 renal macrophages (**Figure 5A**), consistent with a previous report (19). The kidney from heterozygous *Cx3cr1* ^GFP/+^ mice had macrophage number in the middle of those of wild-type and homozygous mice, indicating that one allele of *Cx3cr1* was insufficient to ensure normal renal macrophage density (**Figure 5A**). However, unexpectedly, the number of R2 classical monocytes was normal in the kidneys of *Cx3cr1* ^GFP/GFP^ and *Cx3cr1* ^GFP/+^ mice, implying that monocyte infiltration was unaffected by their loss of CX3CR1 (**Figure 5B**). To further explore the role of CX3CR1 in the establishment of renal macrophages and to understand whether the reduction of renal macrophages under CX3CR1 deficiency is due to an autonomous effect on myeloid cells, we performed competitive BM transplantation by reconstituting lethally irradiated CD45.1 mice with a chimeric 3:1 BM mix from *Cx3cr1* ^GFP/GFP^ and *Cx3cr1* ^+/+^ donors (both in a background of CD45.2) (**Figure 5C**). Two months after transplantation, monocytes in the peripheral blood of the recipients had a ratio about 3:1 between *Cx3cr1* ^GFP/GFP^ and *Cx3cr1* ^+/+^ donor background (**Figure 5D**). Interestingly, when the renal mononuclear phagocytes were examined, the ratios between *Cx3cr1* ^GFP/GFP^ and *Cx3cr1* ^+/+^ cells of classical monocytes (R2), nonclassical monocytes (R4) and macrophages (R7)were 3:1, 3:5 and 1:25, respectively, strongly indicating that CX3CR1 is critical in establishment of renal macrophages from monocytes.

To consolidate the conclusion that CX3CL1-CX3CR1 signaling could facilitate the differentiation of monocyte to macrophages, we first co-cultured classical monocytes from BM with renal epithelial cells line HK-2. Co-incubation of monocytes with renal epithelial cells facilitated their obtainment of renal macrophage phenotype, as attested by their upregulation of renal macrophage markers MHC-II and F4/80 and downregulation of classical monocyte marker CD62L (**Figure 5E**). However, these developmental trends could be significantly slowed by CX3CR1 blockade. The effects of CX3CR1 blockade was also observed when monocytes were co-incubated with primary tubular epithelial cells collected from murine kidney (see Materials and Methods) (**Figure S6A**). To substantiate a direct effect of CX3CL1 on monocyte-to-macrophage differentiation, we supplemented recombinant CX3CL1 to monocyte culture. However, soluble CX3CL1 had no effects on the expression changes in MHC-II, F4/80 or CD62L (**Figure 5F**, upper). Unlike most of other chemokines, CX3CL1 mainly exists as a membrane-tethered form (42). Therefore, we hypothesized that an anchored form of CX3CL1 is required for its action on monocyte differentiation. Indeed, when monocytes were seeded into high affinity-to-protein plates pre-coated with CX3CL1, their differentiation to macrophages was expedited relative to the cells seeded into plates pre-coated with bovine serum albumin (**Figure 5F**, lower). In line with the notion that an anchored form of CX3CL1 is required for monocyte-to-macrophage differentiation, co-culturing monocytes with primary renal epithelial cells in a trans-well system did not promote the obtaining of macrophage phenotype by monocytes (**Figure S6B**). Collectively, we conclude that CX3CL1-CX3CR1 ligation is indispensable for renal macrophage differentiation and this action requires direct interaction between monocytes and renal tissue cells.

## Discussion

In this study, we developed a strategy to successfully discriminate renal mononuclear phagocytes. In particular, we clarified that the majority of myeloid cells in steady state are macrophages by their highest expression of CD64, unaffectedness by Flt3 blocker treatment and negative in Zbtb46 expression. Moreover, the non-DC identity of SM-MØ is further confirmed by the fact that DCs are derived from the BM with a high turnover rate (11), so far, only tissue resident macrophage but not DCs in adult mice have been found containing subtypes with embryo origin and self-maintaining. That the majority of renal F4/80^+^ cells are macrophages is also supported by a recent single cell RNA-seq report (18). It was recently reported that there exist two populations of interstitial macrophages, *i.e*. Lyve1^lo^MHC-II^hi^CX3CR1^hi^ and Lyve1^hi^MHC-II^lo^CX3CR1^lo^, in multiple tissues including lung, heart, fat and dermis (43). Both populations were continuously repopulated by BM-derived monocytes in adult mice. However, our findings of kidney were inconsistent with this interstitial macrophage dichotomy in that all renal macrophages are MHC-II^hi^CX3CR1^hi^. Thus, whether there is a universal signature for sympathetic nerve-associated macrophages across tissue and species is still questionable. The pioneer cross-tissue transcriptome studies established the basis for the molecular signatures of tissue macrophages and DCs (12, 13). However, kidney macrophages were not analyzed in the study (12), and the markers used to purify renal CD11b^+^ DCs could not distinguish from parenchyma nonclassical monocytes in the kidney (13). Future transcriptome analysis on the renal macrophages and renal cDC subsets based on the flow cytometry strategy established in the current study is necessary to supplement the understanding of the generality and specificity of tissue macrophages and DCs.

By examining previously overlooked renal monocyte subsets, we demonstrated that adaptation to renal niches by BM-derived macrophages is a stepwise process. Entering classical monocytes appear to traverse *via* a nonclassical monocyte stage into resident macrophages. Furthermore, by exploiting the roles of CX3CR1, we have advanced the monocyte to renal macrophage transition map. We show that CX3CL1-CX3CR1 signaling is required for the differentiation from monocytes to renal macrophages, so this fractalkine signaling is critical in the establishment of BM-derived macrophages in the kidney. This mechanism may at least partly explain previous studies showing that CX3CR1 deficiency or blockade alleviated inflammation and fibrosis in ischemia-reperfusion injury (44), hypertensive interstitial fibrosis (45) and crescentic glomerulonephritis (21); also, susceptibility of CX3CR1-deficient mice to renal Candida albicans infection (46) and to sepsis-induced kidney damage (47) could be due to a defect of macrophage formation. CX3CL1 exists as a membrane-anchored variant as well as the shed, soluble form (48). The CX3CL1 expressed by vessel endothelial cells serves as an adhesion molecule for monocytes (49). In consistent with our finding that the differentiation of classical monocyte to nonclassical monocytes in the kidney was impaired when CX3CR1 was deficient, nonclassical monocytes highly express CX3CR1 and they particularly depend on CX3CL1 in circulation for homeostasis and survival (50). However, CX3CL1-CX3CR1 interaction does not induce monocyte-macrophage transformation in blood vessel, implying other factors are additionally required for renal macrophage differentiation from monocytes, which is also suggested by our *in vitro* monocyte differentiation experiments: blocking CX3CR1 could only partly abrogate the differentiation of classical monocytes to macrophages in the presence of renal epithelial cells. CX3CR1 is a G protein-coupled receptor (GPCR). Activation of GPCR has been shown to induce human THP-1 monocytic cell line to a macrophage programming (51). Thus, to what extent CX3CR1 promotes monocyte-to-macrophages differentiation is worth future investigation.

## Data Availability Statement

The original contributions presented in the study are included in the article/**Supplementary Material**. Further inquiries can be directed to the corresponding author.

## Ethics Statement

The animal study was reviewed and approved by Institutional Animal Care and Use Committee at Zhejiang University.

## Author Contributions

XS had the conceptualization of the study. XS, PS and QZ designed the experiments with intellectual input from XL and FH. XS supervised the study. QZ, JH and YC performed most of the experiments. WN facilitated confocal microscopy and performed kidney ischemia-reperfusion model. PS and XS wrote the manuscript which was further edited by XL and FH. All authors contributed to the article and approved the submitted version.

## Funding

This work was supported by grants from the National Natural Science Foundation of China (31970898 and 32170894 to XS, 81700365 and 31771266 to PS) and the Primary Research and Development Plan of Zhejiang Province (2020C03034 to FH).

## Conflict of Interest

The authors declare that the research was conducted in the absence of any commercial or financial relationships that could be construed as a potential conflict of interest.

## Publisher’s Note

All claims expressed in this article are solely those of the authors and do not necessarily represent those of their affiliated organizations, or those of the publisher, the editors and the reviewers. Any product that may be evaluated in this article, or claim that may be made by its manufacturer, is not guaranteed or endorsed by the publisher.
