# Supplementary material for: Analysis of Mononuclear Phagocytes Disclosed the Establishment Processes of Two Macrophage Subsets in the Adult Murine Kidney
Source: Front Immunol. 2022 Mar 10;13:805420. doi: 10.3389/fimmu.2022.805420 (PMC8960422; doi:10.3389/fimmu.2022.805420)
Supplement: Supplementary file 1 [file DataSheet_1.docx]

Supplementary Material

## Supplementary Figures

##
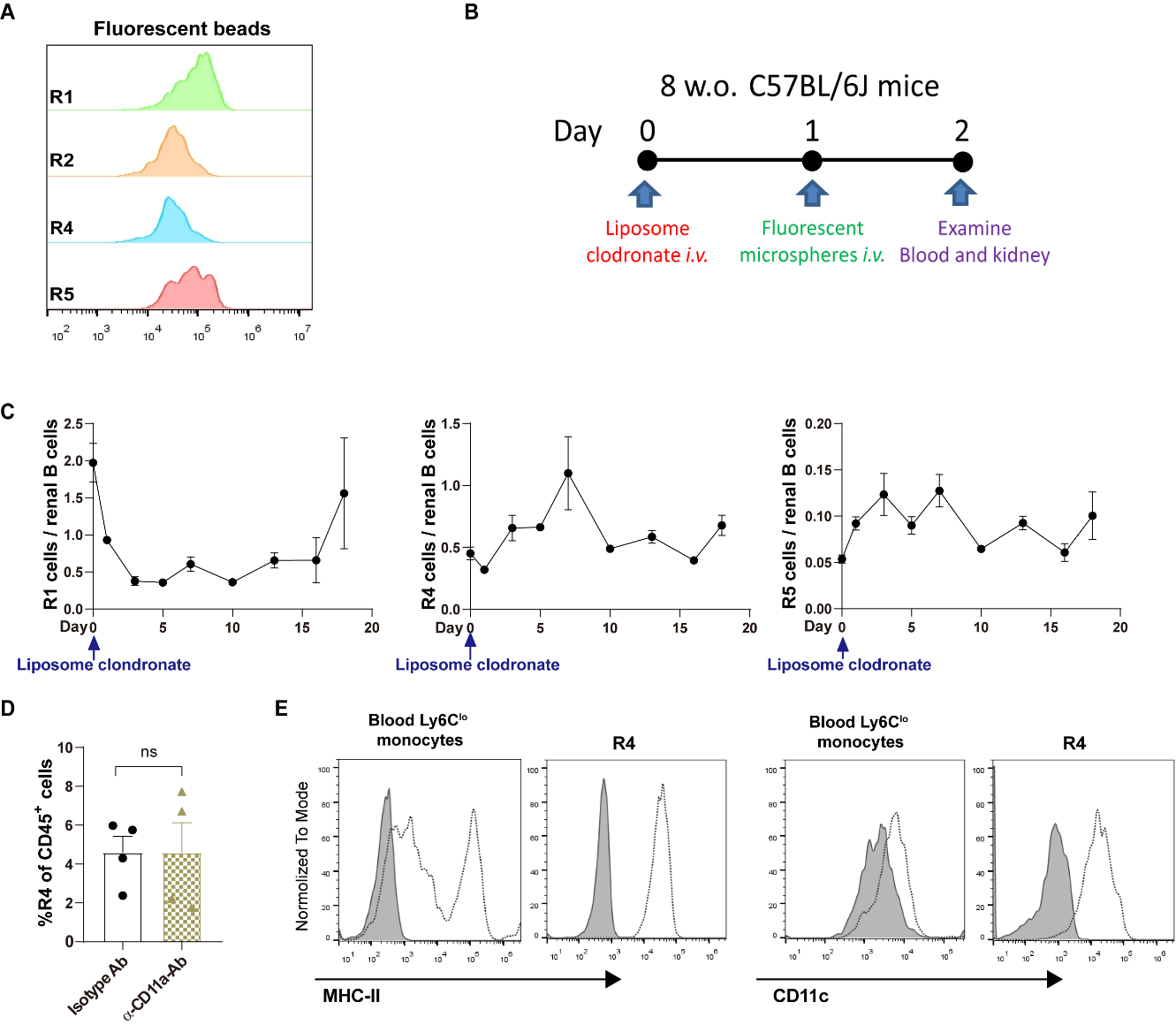


## Supplementary Figure 1. The time-course patterns of R1, R4 and R5 cells after liposome clondronate treatment. n=3-6 for each time point.

##
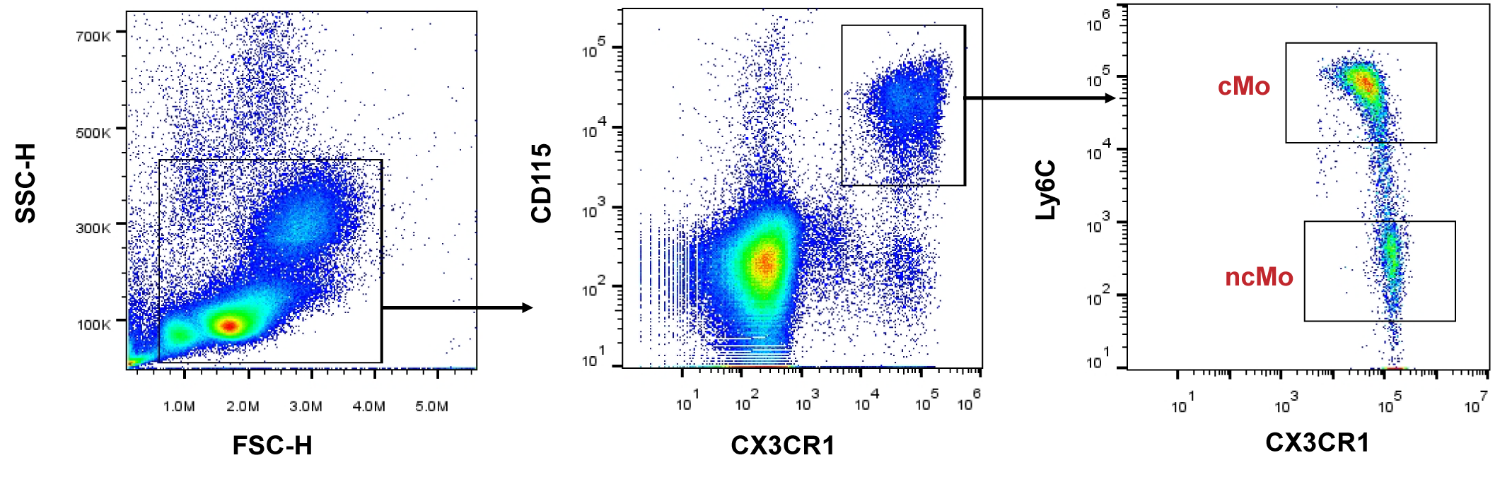


## Supplementary Figure 2. The gating strategy for analyzing blood monocyte subsets. cMo, classical monocytes; ncMo, classical monocytes.

**
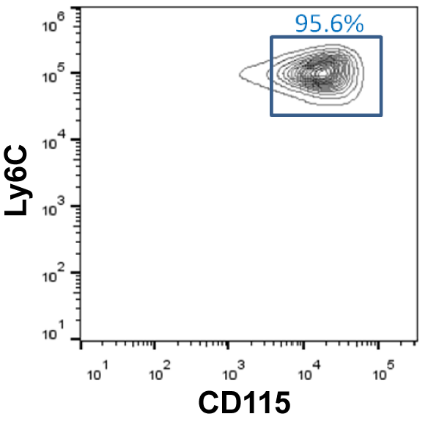
**

**Supplementary Figure 3. The purity of Ly6C^hi^ classical monocytes collected from BM.**


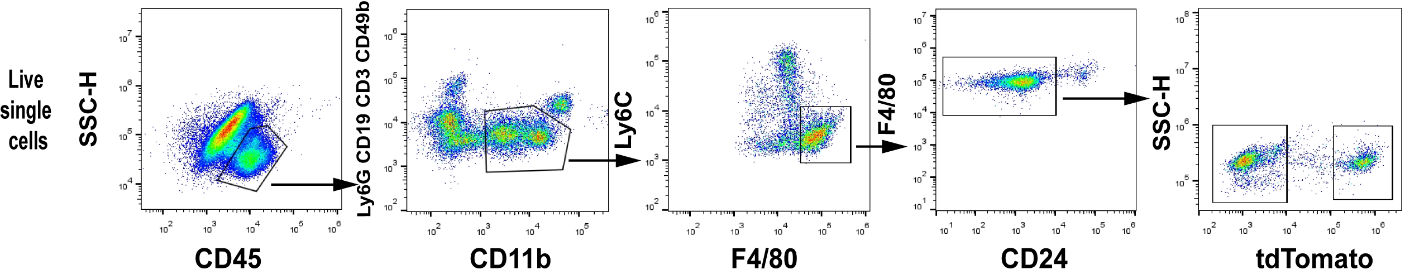


**Supplementary Figure 4. The gating strategy for analyzing tdTomato^―^and tdTomato^+^ macrophages.**


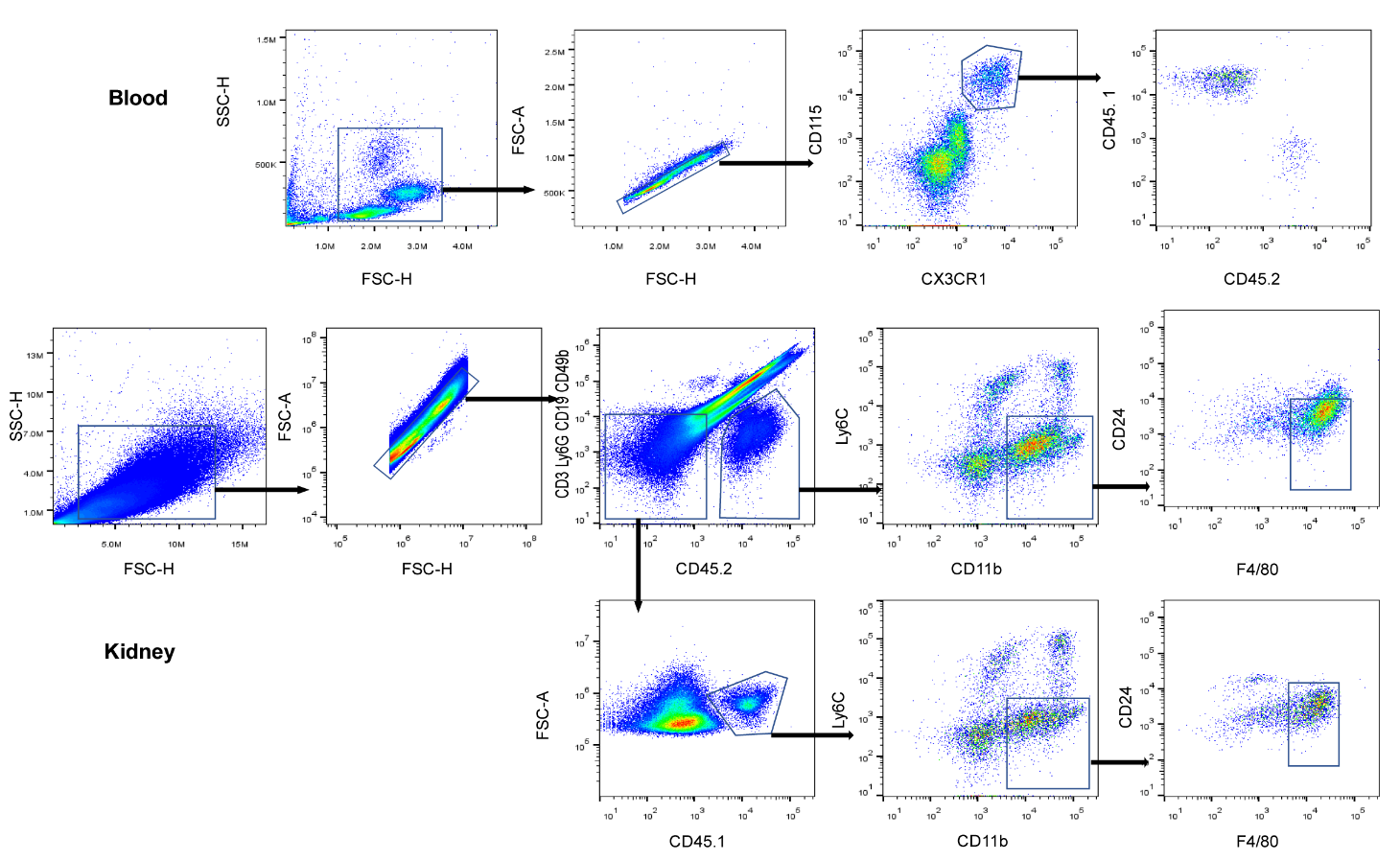


**Supplementary Figure 5. The gating strategies for analyzing the blood (upper panels) and kidney (lower panels) of the CD45.2^+^** **mice after receiving torso-shielded irradiation and bone marrow transplantation from CD45.1^+^ donors.**


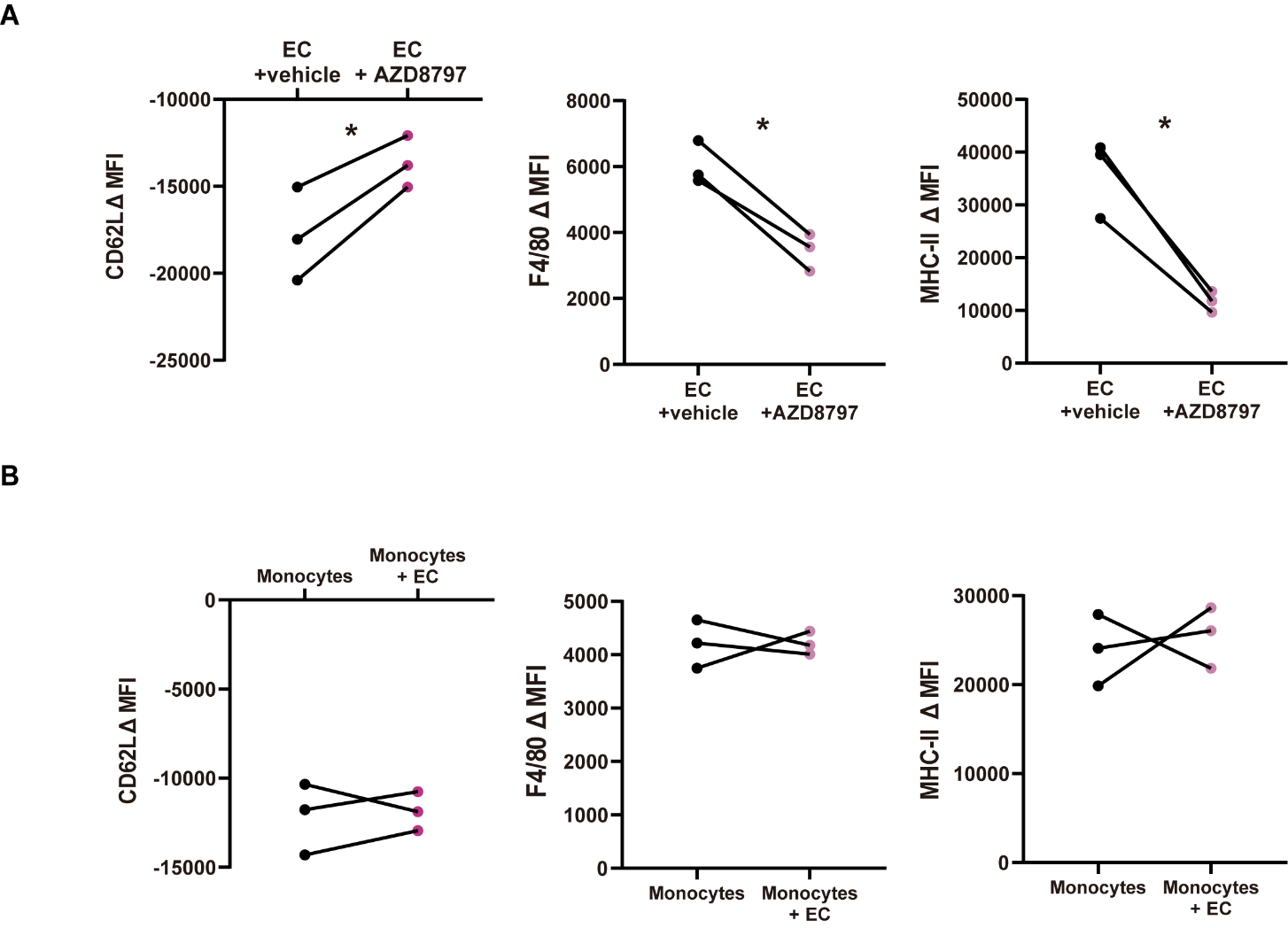


**Supplementary Figure 6. The ligation of monocytic CX3CR1 with an anchored form of CX3CL1 facilitated differentiation to macrophages.** The changes of mean fluorescent intensities (ΔMFI) of surface CD62L, F4/80 and MHC-II on classical monocytes after *ex vivo* culture. (**A**) Monocytes were co-incubated with primary murine tubular epithelial cells in the presence of vehicle or AZD8797, a CX3CR1 blocker. (**B**) Monocytes were co-incubated with or without primary murine tubular epithelial cells in a trans-well system. *P* values were calculated using a two-tailed paired t-test. **P* < 0.05.
